# The Role of Church Support Networks in the Relationship between Discrimination and Psychiatric Disorders among Older African Americans

**DOI:** 10.1007/s13644-021-00464-z

**Published:** 2021-07-15

**Authors:** Ann W. Nguyen, Fei Wang, Weidi Qin, Tyrone C. Hamler

**Affiliations:** Jack, Joseph and Morton Mandel School of Applied Social Sciences, Case Western Reserve University

**Keywords:** social support, church relationships, older African Americans, mental health

## Abstract

**Background:**

Few studies have examined the effects of discrimination on mental health specifically among older African Americans despite it being a common experience in this population. Further, knowledge on social resources, such as social relationships, that could mitigate the effects of discrimination is limited in this population. Given the historical and contemporaneous importance of the Black church in African American communities, church members are important support network members and a major source of social support for older African Americans.

**Purpose:**

To address these knowledge gaps, this study will (1) examine the association between racial discrimination and psychiatric disorders; and (2) determine whether church relationships moderate the impact of racial discrimination on psychiatric disorders.

**Methods:**

Data from African American respondents aged 55 and older were drawn from the National Survey of American Life (*N* = 837). Church relationship variables included receipt of emotional support from, frequency of contact with, and subjective closeness to church members. Regression analyses were used to test the study aims.

**Results:**

Analyses indicated that more frequent experiences of racial discrimination were associated with meeting criteria for any DSM-IV disorder and a greater number of DSM-IV disorders. Significant interactions revealed that frequency of contact with and subjective closeness to church members mitigated the association between discrimination and meeting criteria for any 12-month disorder and number of 12-month disorders.

**Conclusions and Implications:**

Altogether, these findings support the literature on the detrimental effects of discrimination on the mental health of older African Americans and provide a more nuanced understanding of the role of church members in the lives of older African Americans. The study findings suggest that church relationships are effective stress coping resources for older African Americans dealing with discrimination. Given the importance and relevance of church members, initial clinical assessments should assess clients’ level of religious involvement and relationships with church members.

## Introduction

Experiences of racial discrimination are pervasive in the lives of African Americans. Instances of institutional as well as interpersonal discrimination are prevalent, indicating that discrimination extends across multiple life domains (National Public Radio et al. 2017). Findings from a recent national survey indicated that over one in two African Americans have experienced racial discrimination in their interactions with police, when applying for jobs, and being paid equally or considered for promotions (National Public Radio et al. 2017). At the interpersonal level, most African Americans in the survey reported having personally experienced racial slurs. Over half of African Americans surveyed said that people have made negative assumptions or insensitive or offensive comments about their race, and two in five have experienced racial violence. These experiences have led many African Americans to avoid calling the police and to forgo medical care even when necessary to avoid potential exposure to discrimination. Given its prevalence, pervasiveness, and consequences, racial discrimination constitutes a chronic stressor for African Americans and directly threatens the mental and physical health of this population.

Although older African Americans are less likely to report experiences of discrimination than their younger counterparts, it, nevertheless, is a common experience in this population. Yet, there is a dearth of systematic investigations of discrimination, its mental health consequences, and factors that mitigate the harm of discrimination among older African Americans. A better understanding of discrimination among older African Americans is critical, as it is projected that by 2030, one in five Americans will be aged 65 and older, and the older adult population will be more racially and ethnically diverse (U. S. Census Bureau 2015). Among older persons, African Americans are a particular group of interest because they belong to a cohort of Americans that have endured government-sanctioned racial discrimination (e.g., Jim Crow, redlining) and more implicit forms of discrimination. These repeated exposures to discrimination over the life course represents cumulative risk for mental health problems. The present analysis investigates the association between racial discrimination and psychiatric disorders among older African Americans and whether relationships with church members moderate this association.

### Discrimination and Mental Health

National data indicate that less than one fifth of African Americans (17%) met criteria for a psychiatric disorder in the past 12 months (Vilsaint et al. 2019), and over one third of African Americans (37%) met criteria for a psychiatric disorder over their lifetime (Alvarez et al. 2019). The most common types of disorders in this population are anxiety and mood disorders (Vilsaint et al. 2019). The 12-month prevalence rates for anxiety and mood disorders among African Americans are 12% and 7%, respectively (Vilsaint et al. 2019), and the lifetime prevalence rates of anxiety and mood disorders among African Americans are 22% and 14%, respectively (Alvarez et al. 2019). Among anxiety disorders, social anxiety disorder and posttraumatic disorder are the most prevalent (Himle et al. 2009), and major depressive disorder is the most common mood disorder diagnosed in African Americans (Breslau et al. 2006; Hasin et al. 2018).

Previous research has established discrimination as a risk factor for psychiatric disorders and other mental health problems (Taylor and Chatters 2020). Research on discrimination among African Americans indicates that experiences of discrimination are associated with mood and anxiety disorders, psychological distress, and hopelessness (Head and Thompson 2017; McLaughlin et al. 2010; Pieterse et al. 2012; Chae et al. 2011; Mitchell et al. 2020). More specifically, studies in this area have identified associations between discrimination and social anxiety disorder (Levine et al. 2014) and generalized anxiety disorder (Soto et al. 2011). Discrimination is also positively associated with depressive symptoms cross-sectionally and longitudinally (Schulz et al. 2006; Britt-Spells et al. 2016; Qin et al. 2020; White et al. 2020). Longitudinal studies have found that increases in experiences of discrimination over time is associated with increases in depressive symptoms over time (Schulz et al. 2006; Qin et al. 2020).

Research on older African Americans has similarly demonstrated the detrimental effects of discrimination on mental health. Mouzon et al. (2016) examined the relationship between everyday discrimination and mental health (mood and anxiety disorders, depressive symptoms, psychological distress) among older African Americans and found that racial and non-racial everyday discrimination were associated with worse mental health. Empirical evidence also indicates that discrimination is positively associated with serious psychological distress (Nguyen et al. 2017) and generalized anxiety disorder (Nguyen 2018) among older African Americans. Collectively, evidence on the effects of discrimination is unequivocal; it indicates that discrimination is detrimental to the mental health of African Americans across the life course and is a risk factor for some psychiatric disorders. Information on factors that can moderate the effects of discrimination is imperative for addressing this ubiquitous social problem.

### Church Support and Mental Health

Social support is an important stress coping resource. Numerous studies indicate that strong social support networks are effective in reducing the risk for mental health problems, such as depressive symptoms and psychological distress (Lincoln et al. 2003, 2005; Lincoln et al. 2012). Specifically, African Americans who receive frequent support from their families are less likely to be diagnosed with depression (Lincoln and Chae 2012), experience fewer depressive symptoms (Lincoln et al. 2005), and report lower levels of psychological distress (Lincoln et al. 2003; Dilworth-Anderson et al. 1999). In addition to depression, social support can protect against other psychological problems. Empirical findings demonstrate that receiving more social support is associated with a decreased risk for suicidality (Lincoln et al. 2012) and lower odds of meeting criteria for social anxiety disorder (Levine et al. 2015) and post-traumatic stress disorder (Nguyen et al. 2016). Together, these studies indicate that social relationships can protect against a range of psychiatric problems and are important social resources for African Americans.

Historically, the Black Church has played a prominent role in African American communities. In addition to being a religious institution, frequently, it is at the center of social, civic, and political life in African American communities (Lincoln and Mamiya 1990). The Black church is of particular importance for older African Americans, who have higher rates of service attendance and religious participation than younger African Americans and older Whites (Taylor et al. 2014). Consequently, church members are important support network members and a major source of social support for older African Americans. The Black church is particularly well positioned to help African Americans deal with discrimination. As an institution that is primarily founded, financed, and controlled by African American communities, it provides African Americans opportunities to learn organizational skills and gain leadership positions that would have been difficult to access within the broader society due to disenfranchisement (Myrdal 1944). Further, integration within a religious community that shares similar values and beliefs that reinforces group (and ethnic) identity can enhance African Americans’ sense of self-worth (Mays and Nicholson 1933). Sermonic traditions based in liberation and defiance theology are also unique to the Black church and provides a spiritual framework for coping with discrimination (Frazier and Lincoln 1974).

Research has found that church relationships can protect against a range of mental health problems (Nguyen 2020). Studies indicate that church support is negatively associated with depression, depressive symptoms, and suicide ideation and attempts (Chatters et al. 2015; Krause and Wulff 2005; Nooney and Woodrum 2002; Chatters et al. 2011). Given the protective effects of church support, an emerging area of research has examined how church relationships may mitigate the harmful effects of chronic stressors, such as discrimination. Similar to research on the moderating effects of service attendance on the relationship between discrimination and mental health problems (Bierman 2006; Ellison et al. 2008), research on church support indicates that this type of support can also moderate the discrimination-mental health association. For example, Nguyen (2018) found that church relationships can buffer against the deleterious effects of discrimination on mental health among older African Americans. In particular, this study found that frequency of contact with and subjective closeness to church members buffered against the effects of discrimination on generalized anxiety disorder (GAD). Among respondents who had low levels of contact with and subjective closeness to church members, the odds of meeting criteria for GAD increased as experiences of discrimination increased. However, among respondents who had high levels of contact with and subjective closeness to church members, experiences of discrimination and GAD were unrelated. Similarly, Ellison et al. found that church support buffered against the harmful effects of experiences of major discrimination on depression and life satisfaction among African Americans (2017). That is, among respondents who infrequently received support from church members, more frequent experiences of discrimination were associated with more depressive symptoms and lower life satisfaction. In contrast, discrimination was unrelated to either depressive symptoms or life satisfaction among respondents who reported frequently receiving church support. Contrary to the studies previously discussed, another study by Nguyen et al. (2017) did not find a mitigating effect for church support in the association between discrimination and psychological distress among African American men. Study findings showed that discrimination was predictive of higher levels of psychological distress, especially for men who reported receiving more emotional support from church members, indicating a resource mobilization effect. Overall, research on church relationships has underscored its protective qualities. Although findings related to the moderating effects of church support has been equivocal, there is substantial evidence suggesting that it can attenuate the deleterious effects of chronic stressors, such as discrimination, on mental health.

### Focus of the Study

The bulk of research on discrimination among African Americans has focused on adults across the life span, with few focusing specifically on older African Americans despite it being a common experience in this population. Further, only a handful of investigations have examined how church relationships moderate the association between discrimination and mental health, and these studies have mostly focused on a narrow range of mental problems. This has resulted in a critical knowledge gap in the psychiatric effects of discrimination among older African Americans and moderating factors. Further, few studies examine multiple aspects of church relationships when investigating their moderating effects. As a result, little is known about how other church relational aspects (e.g., contact, subjective closeness) may interact with discrimination to influence mental health.

To address these knowledge gaps, this study will examine the association between racial discrimination and 12-month DSM-IV psychiatric disorders and whether church relationships moderate this association. Specifically, we will examine the moderating effects of frequency of contact with, emotional support from, and subjective closeness to church members in a nationally representative sample of older African Americans. This analysis will contribute to the discrimination literature by 1) examining a broader range of psychiatric disorders as well as the number of psychiatric disorders; 2) including multiple aspects of church relationships as moderators; and 3) focusing specifically on older African Americans using a nationally representative sample.

## Methods

### Sample

The National Survey of American Life: Coping with Stress in the 21st Century (NSAL) was collected by the Program for Research on Black Americans at the University of Michigan’s Institute for Social Research. The field work for the study was completed by the Institute for Social Research’s Survey Research Center, in cooperation with the Program for Research on Black Americans. The NSAL featured a national multistage probability design consisting of 64 primary sampling units involved 6,082 face-to-face interviews with individuals aged 18 or older (for a more detailed discussion of the NSAL sample, see Jackson et al., 2004). This study used the subsample of African Americans age 55 and older from the NSAL. After listwise deletion of cases due to missing data, the analytic sample featured 837 older African Americans. Listwise deletion is considered acceptable and has little impact on the validity of statistical inferences if missing data represents less than 10% of the sample.

### Measures

#### Independent variables.

*Frequency of contact with church members* was assessed with the following question, “How often do you see, write or talk on the telephone with members of your church? Would you say nearly every day (6), at least once a week (5), a few times a month (4), at least once a month (3), a few times a year (2), or never (1)?” *Subjective closeness to church members* was assessed by the question, “How close are you to the people in your church? Would you say very close (4), fairly close (3), not too close (2), or not close at all (1)?” *Everyday racial discrimination* was measured with a summary score of 10 items developed by Williams, Yu, Jackson, and Anderson (1997) that assess episodes of unfair treatment experienced during the past 12 months (Cronbach’s alpha = .90). Response categories ranged from never (0) to almost everyday (5). After each item, respondents were asked the reason for why they experienced the unfair treatment (e.g., ancestry/national origins, gender, race, age, skin tone). Respondents who attributed the unfair treatment to race, ancestry/national origins, or skin tone were classified as having experienced racial discrimination in that episode of unfair treatment.

#### Dependent variables.

The two dependent variables in this analysis—*any 12-month DSM-IV disorder* and *number of 12-month DSM IV disorders*—were assessed using the DSM-IV World Mental Health Composite International Diagnostic Interview (WMH-CIDI). The WMH-CIDI is a fully structured diagnostic interview (Kessler and Ustün 2004). Any 12-month disorder includes panic disorder, social phobia, agoraphobia without panic, generalized anxiety disorder, major depressive disorder, dysthymia, bipolar disorder I, bipolar disorder II, and subthreshold bipolar disorder (bipolar disorders counted as one disorder). The number of 12-month DSM-IV disorders is a count of all previously mentioned disorders.

#### Control variables.

Multivariate analysis controlled for gender, age, education, family income, marital status, region, religious service attendance, and chronic health conditions. Gender was dummy coded. Age, education, family income, service attendance, and chronic health conditions were scored continuously; age and education were assessed in years. Marital status was coded to differentiate respondents who were married or cohabiting; separated, divorced, or widowed; and never married. Region was coded to distinguish between the South, Northeast, North Central, and West. Family income was coded in dollars. Due to its skewed distribution, we used the log of family income. Missing data for family income and education were imputed using an iterative regression-based multiple imputation approach incorporating information about age, sex, region, race, employment status, marital status, home ownership, and nativity of household residents.

### Analysis Strategy

**Figure 1.**
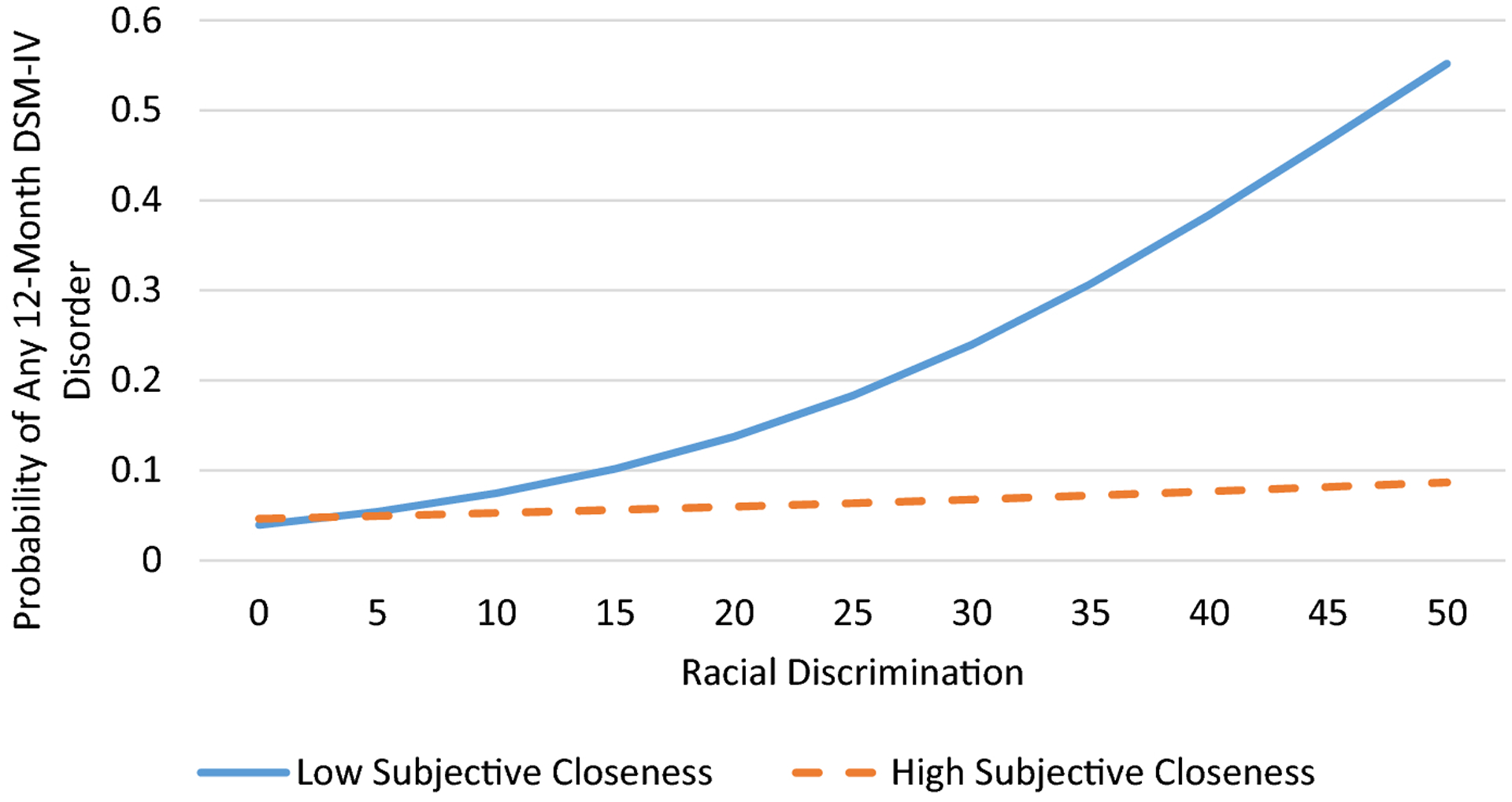
Predicted probability of meeting criteria for any 12-month DSM-IV disorder by racial discrimination and subjective closeness to church members among older African American respondents.

**Figure 2.**
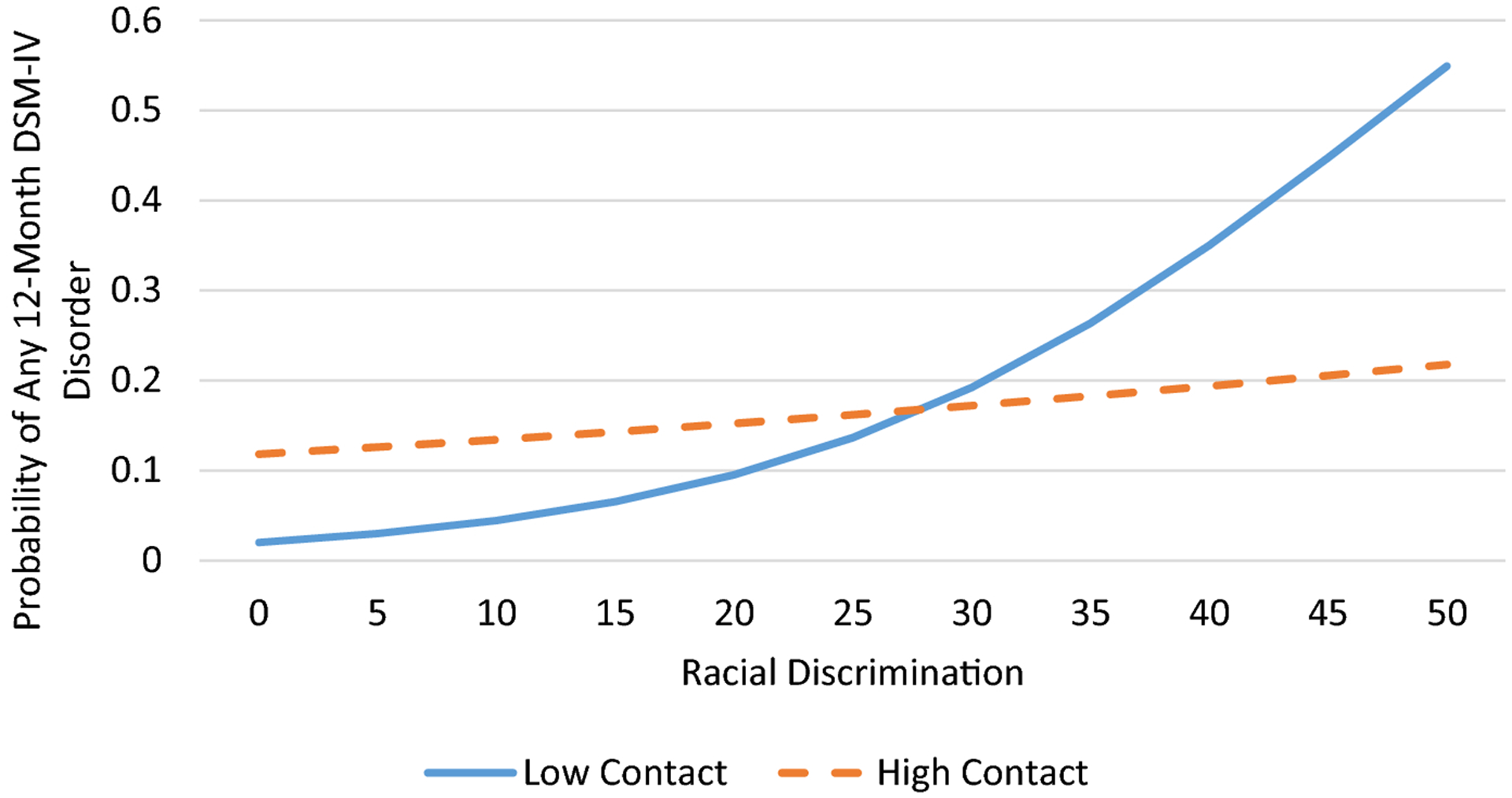
Predicted probability of meeting criteria for any 12-month DSM-IV disorder by racial discrimination and frequency of contact with church members among older African American respondents.

Logistic regression was used to determine the associations between discrimination, church relationships, and any 12-month DSM-IV disorder. According to Mood (2010), odds ratios are sometimes difficult to interpret because they reflect unobserved heterogeneity across samples, groups within samples (in the case of interaction effects), and points in time. Thus, we present both adjusted odds ratios and average marginal effects (AMEs) in Table 2, which presents results from the logistic regression analyses. The AME represents the average effect of x on the probability of y=1. In other words, the AME is the average change in the probability of y=1 when x increases by one unit. Due to the nonnormal distribution of the 12-month DSM-IV disorder count variable, we used negative binomial regression to examine the associations between discrimination, church relationships, and number of 12-month DSM-IV disorders. We used interaction terms between discrimination and emotional support, frequency of contact, and subjective closeness to test the moderating effects of church relationships on the association between discrimination and DSM-IV disorders. We presented four regression models for each outcome; we estimated the association between discrimination and 12-month DSM-IV disorders in Model 1. In Models 2 through 4, we individually tested the interactive effects discrimination and church relationships (i.e., emotional support, frequency of contact, subjective closeness) on DSM-IV disorders. To illustrate significant interactions, we plotted the estimated values for number of 12-month DSM-IV disorders and predicted probabilities of any 12-month DSM-IV disorder (Figures 1–2). Subjective closeness to and frequency of contact with church members were portrayed as dichotomized variables in the interaction plots. Low and high subjective closeness and frequency of contact groups were represented by respondents with subjective closeness and frequency of contact scores of 1 standard deviation below and above the mean, respectively. All multivariate analyses took into account the complex multistage clustered design of the NSAL sample, unequal probabilities of selection, nonresponse, and poststratification.

## Results

Table 1 presents demographic characteristics of the sample and distribution of study variables. The mean age of respondents was 68 years. Women (60%) comprised a greater proportion of the sample than men. On average, respondents reported a little over 11 years of formal education and an average family income of $27,652. Slightly over half of the sample were either separated, divorced, or widowed, and close to 40% were either married or cohabiting. The majority of the sample (56%) resided in the South. Overall, respondents reported high levels of frequency of contact with and subjective closeness to church members. The mean number of chronic health conditions reported was 2.6. About 6% of the sample met criteria for a DSM-IV disorder in the past 12 months. The mean racial discrimination score for the sample was 5.7.

Logistic regression analysis indicated that racial discrimination was associated increased odds of meeting criteria for any 12-month DSM disorder (Table 2, Model 1). Tests for the moderating effects of church relationships (Models 2–4) showed that subjective closeness to and frequency of contact with church members mitigated the effects of discrimination on meeting criteria for a DSM-IV disorder. The significant interaction between subjective closeness and discrimination indicated that while discrimination was unrelated to any DSM-IV disorder among respondents who reported high levels of subjective closeness, among respondents who reported low subjective closeness, more frequent experiences of discrimination was associated with an increased probability of meeting criteria for a DSM-IV disorder (Figure 1). The significant interaction between frequency of contact and discrimination indicated that discrimination was positively associated with any 12-month DSM-IV disorder for all respondents (Figure 2). However, the magnitude of this association was substantially stronger among those who reported low contact with church members than those who reported high contact with church members.

**Figure 3.**
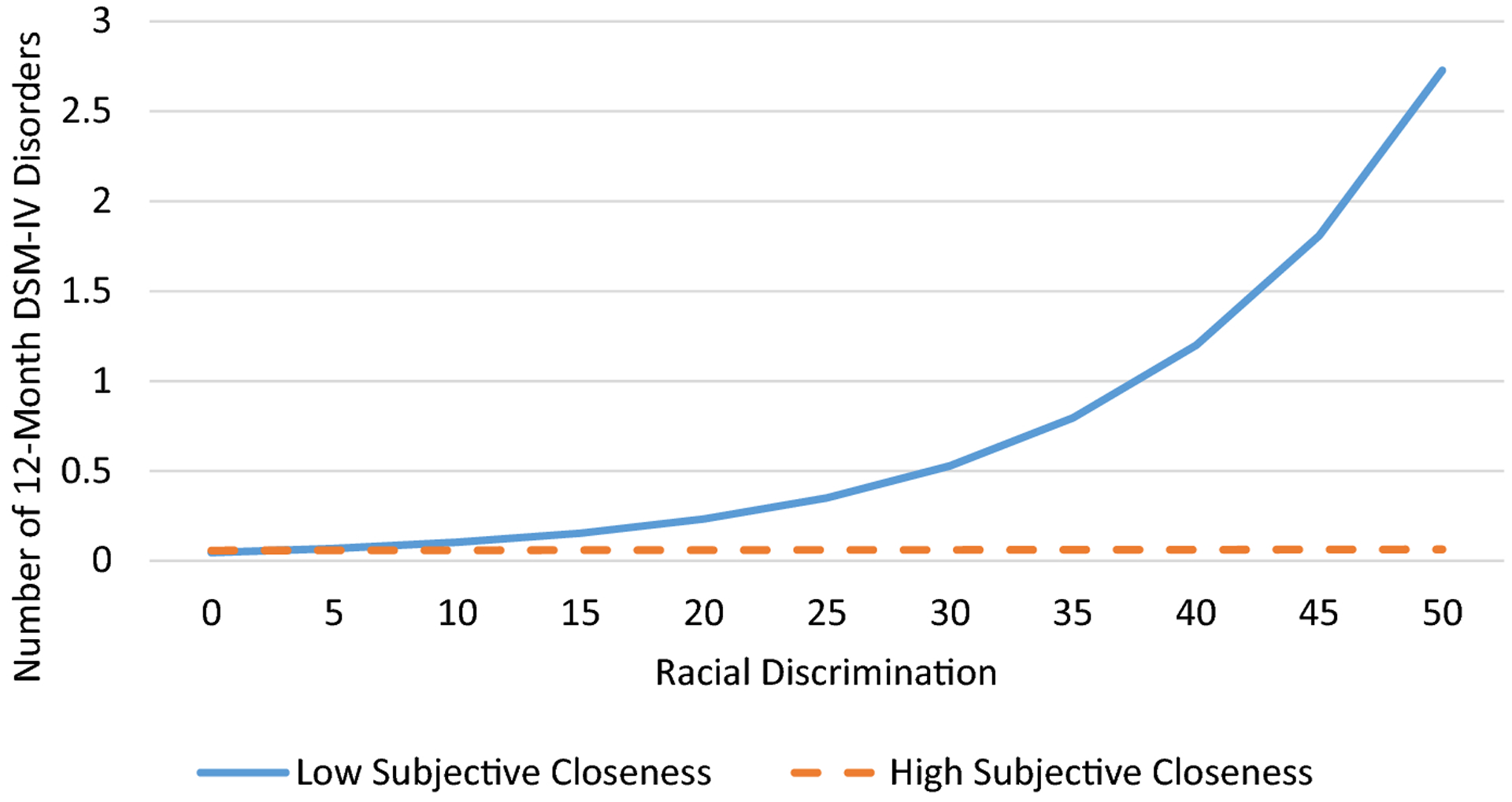
Predicted value of number of 12-month DSM-IV disorders by racial discrimination and subjective closeness to church members among older African American respondents.

**Figure 4.**
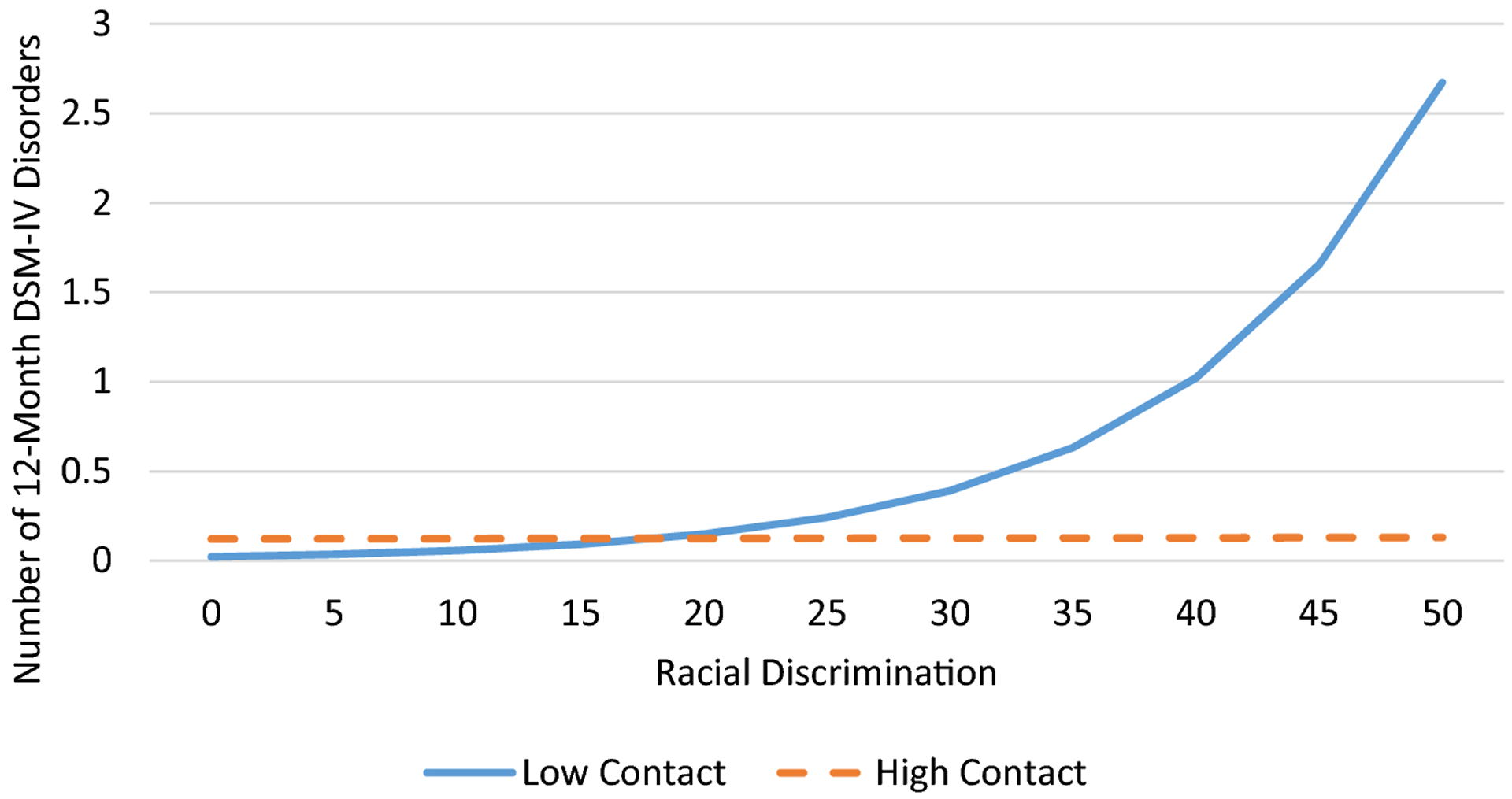
Predicted value of number of 12-month DSM-IV disorders by racial discrimination and frequency of contact with church members among older African American respondents.

Results from the negative binomial regression analysis (Table 3) indicated that racial discrimination was positively associated with number of DSM-IV disorders (Model 1); that is, respondents who reported more frequent experiences of racial discrimination met criteria for a greater number of DSM-IV disorders. Tests for the moderating effects of church relationships (Models 2–4) revealed that subjective closeness to and frequency of contact with church members attenuated the association between discrimination and number of DSM-IV disorders. Specifically, the significant interactions indicated that among respondents who had high levels of subjective closeness to (Figure 3) or contact with (Figure 4) church members, discrimination was unrelated to number of DSM-IV disorders. In contrast, among respondents who had low levels of subjective closeness to or contact with church members, discrimination was positively associated with number of DSM-IV disorders.

## Discussion

The current analysis investigated the moderating role of church support in the association between everyday racial discrimination and psychiatric disorders in a nationally representative sample of older African Americans. The analysis examined a range of church relationship characteristics, including frequency of contact with church members, subjective closeness to church members, and receiving emotional support from church members. The findings indicated that more frequent experiences of racial discrimination were associated with meeting criteria for any DSM-IV disorder and a greater number of DSM-IV disorders in the past 12 months. This is consistent with the well-established literature documenting the psychological harm of discrimination. Research in this area has shown that discrimination is predictive of serious psychological distress (Nguyen et al. 2017), mood disorders (Clark et al. 2015), generalized anxiety disorder (Clark et al. 2015), and social anxiety disorder (Levine et al. 2014), among other mental health conditions.

Further, the findings indicated that church support can attenuate the association between racial discrimination and psychiatric disorders. Specifically, frequency of contact with and subjective closeness to church members mitigated the relationship between racial discrimination. Among older African Americans who had low subjective closeness to church members, as reported instances of racial discrimination increased, so did the probability of meeting criteria for any psychiatric disorder and the number of psychiatric disorders. However, among older African Americans who had high subjective closeness, racial discrimination was not associated with the number of DSM-IV disorders. This indicates that higher subjective closeness to church members can be beneficial for older African Americans, especially as a stress coping resource.

Although our analysis demonstrated that contact with church members fully mitigated the relationship between discrimination and number of psychiatric disorders, contact only partially attenuated the relationships between discrimination and the probability of meeting criteria for any psychiatric disorder. That is, discrimination was associated with a greater likelihood of meeting criteria for any 12-month DSM-IV disorder for all respondents; however, this association was substantially weaker among respondents who reported high levels of contact with church members.

These findings are concordant with studies documenting the stress buffering effects of social relationships and social support. Prior research has identified that frequency of contact with and subjective closeness to church members can buffer against the effects of everyday discrimination on generalized anxiety disorder among older African Americans (Nguyen 2018). Similarly, research on African American adults has indicated that social support from church members can buffer against the effects of major discrimination on depression (Ellison et al. 2017).

The stress buffering hypothesis (Wheaton 1985; Cohen and Wills 1985) may provide an explanation of how church support could moderate the association between discrimination and mental illness. Cognitive appraisals are central to the stress process. When a potentially stressful event occurs and the individual believes that they do not have the necessary resources to cope with the event, this event is appraised as stressful and can lead to negative physiological and psychological sequelae (Lazarus and Folkman 1984). However, if the individual believes that they do in fact have the necessary resources to cope with the event, then this event will not be appraised as stressful (Lazarus and Folkman 1984). Thus, the stress buffering hypothesis posits that social relationships can moderate the association between perceived stress and health by acting as a stress coping resource from which individuals may draw. Specifically, social relationships can intervene in the cognitive appraisal of the stressor by influencing an individual’s perception of the problem. Perceiving that they have adequate support from others to address the stressful event bolsters the individual’s confidence in coping with the stressor. Consequently, with adequate support, the individual would perceive the event to be less stressful or not at all stressful (Cohen and Wills 1985).

In fact, theoretical works on the connection between religion and mental health have suggested that one of the mechanisms by which religious involvement influences mental health is through positive self-perceptions and self-efficacy (Ellison and Levin 1998). Individuals who are religiously involved, especially within their congregations, are likely to gain a greater sense of self-efficacy. This sense of self-efficacy leads to more positive cognitive appraisals of stressors and mitigates the effects of stressors on mental health. Within the context of the current findings, respondents’ high levels of contact with and feelings of subjective closeness to church members may be associated with a greater sense of self-efficacy in dealing with discriminatory events and thus diminishes the relationship between discrimination and psychiatric disorders. Essentially, these aspects (i.e., contact and subjective closeness) of supportive relationships with church members are likely effective stress coping resources for older African Americans that can intervene in the discrimination-mental health connection.

Church relationships are particularly meaningful for older African Americans, and the support provided by church members is distinct from other types of support, such as support from family and friends. Church relationships are social resources that are available only to individuals socially embedded within a religious community that shares similar beliefs and values based on a common faith and religious teachings. Church relationships are among the few relationships, aside from family relationships, that endure throughout a large proportion of the life course, as many individuals remain with the same congregation throughout their lives. Thus, older adults tend to be established members of their congregation (Taylor and Chatters 1988). Further, a number of important milestones over the life course are shared among members of a congregation, such as christenings, marriage, and funerals (Taylor and Chatters 1988; Krause 2006). Given these unique qualities of church relationships, church members play a critical role in the mental health and well-being of older African Americans.

Although church members are considered secondary network members, church relationships are particularly close among older adults (Krause and Hayward 2015). Research has demonstrated the importance of secondary network members who are socially similar to the support recipient, as they are likely to share direct experiential knowledge of the recipient’s stressors (e.g., discriminatory experiences) (Thoits 2011). This direct experiential knowledge of the stressor is important for the provision of effective support and active coping assistance. Experience-based support is important because this type of support is based on a better and more personal understanding of the stressor. Support network members who have directly experienced similar stressors are able to better empathize with the support recipient as well as are able to anticipate their concerns and emotional reactions. This empathic understanding allows the support recipient to express their feelings of distress more freely and receive validation for these feelings and experiences. The combination of being able to more freely express feelings of distress, validation, and empathy reduces physical arousal and can mitigate the negative psychological sequelae of discrimination. Consequently, as subjectively close secondary support network members, church members, who are likely to be socially similar, can provide experience-based support, which can offset the harmful effects of discrimination.

### Limitations

The current findings should be interpreted within the context of the study’s limitations. First, because the NSAL only surveyed community-dwelling adults, the current findings are not generalizable to institutionalized and homeless individuals. Second, all measures in this study were self-reported. Self-reported measures are susceptible to recall and social desirability biases. Third, the findings do not permit for causal inferences given the cross-sectional design of this study. Thus, it is unclear whether experiencing more frequent instances of discrimination resulted in mental illness, or individuals with mental illness were more likely to experience discrimination. Future research using longitudinal study design is necessary to establish temporal ordering in the relationship between racial discrimination and mental illness.

### Implications & Conclusion

Despite these limitations, the current findings can inform practice with and interventions for older African Americans. Given the importance and relevance of church members, initial clinical assessments should assess clients’ level of religious involvement and relationships with church members. In particular, information on objective (e.g., network size, frequency of contact) as well as subjective (e.g., satisfaction with relationships, subjective closeness) relationship qualities should be obtained to help determine the client’s available stress coping resources. Additionally, interventions to increase formal mental health service use among older African Americans should consider the role of church members. These interventions are particularly important, as older African Americans are more likely to face multiple barriers to mental health service use and are less likely to engage mental health services than older non-Hispanic Whites (Nguyen et al. 2020). Interventions to increase formal mental health service use among older African Americans could train select church members, especially those who are socially well-connected within the church and are able to reach a greater number of congregants, to 1) provide psychoeducation on what mental health services include and their benefits; 2) identify available mental health services that are appropriate for individuals experiencing specific mental health problems, and 3) provide guidance on accessing mental health services.

In conclusion, this study extends knowledge on the connection between church-based relationships and mental health and contributes to a limited body of research on the moderating role of church relationships in the association between discrimination and mental health among older African Americans. Although some studies have investigated the stress-buffering effects of church support on mental health, few studies have examined the role of church support as a protective factor among older African Americans. Given the importance of religion among older African Americans and higher rates of religious involvement and church attendance, which are associated with more frequent supportive exchanges with church member, this area of research is particularly relevant to this growing population. An additional strength of this analysis is the examination of both objective (frequency of contact) and subjective (subjective closeness) dimensions of church relationships, which provides a more complete understanding of church relationships and its role in mental health. By examining both dimensions of church relationships, we found that frequency of contact with and subjective closeness to church members functioned differently as moderating factors in the association between discrimination and psychiatric disorders. This suggests that objective and subjective dimensions of church relationships are distinctive and have differing mechanisms in the stress process. Altogether, these findings support the literature on the detrimental effects of racial discrimination on the mental health of African Americans and provide a more nuanced and in depth understanding of the role of church members in the lives of older African Americans. The study findings suggest that church relationships are effective stress coping resources for older African Americans dealing with racial discrimination.

## Figures and Tables

**Table 1.** Demographic Characteristics of the Sample and Distribution of Study Variables

	N (%)	Mean (S.D.)	Min	Max
Age		66.8 (8.60)	55	93
Gender				
Male	300 (40.43)			
Female	537 (59.57)			
Education		11.21 (3.50)	0	17
Family Income		27,652.31 (34,089.94)	0	450,000
Marital Status				
Married/Cohabiting	250 (39.17)			
Separated/Divorced/Widowed	517 (54.07)			
Never Married	60 (6.22)			
Region				
Northeast	108 (15.79)			
North Central	154 (19.31)			
South	525 (55.62)			
West	50 (9.28)			
Chronic Health Conditions		2.64 (1.90)	0	11
Service Attendance		3.28 (1.10)	1	5
Frequency of Contact with Church Members		4.30 (1.64)	1	6
Subjective Closeness to Church Members		3.31 (0.86)	1	4
Racial Discrimination		5.69 (8.67)	0	50
Any 12-Month DSM-IV Disorder				
Yes	54 (6.25)			
No	732 (93.75)			
Number of 12-Month DSM-IV Disorders		0.10 (0.42)	0	3

Percents and N are presented for categorical variables and Means and Standard Deviations are presented for continuous variables. Percentages are weighted and frequencies are un-weighted.

**Table 2.** Logistic regression analysis for any 12-month DSM-IV disorder among older African Americans

	Model 1	Model 2	Model 3	Model 4
	AOR (95% CI)	AOR (95% CI)	AOR (95% CI)	AOR (95% CI)
Racial Discrimination	1.05 (1.01–1.89)*	1.16 (1.06–1.29)**	1.14 (1.06–1.24)**	1.02 (0.90–1.15)
Subjective Closeness	0.81 (0.47–1.41)	1.17 (0.61–2.24)	0.82 (0.47–1.44)	0.81 (0.47–1.39)
Frequency of Contact	1.41 (1.06–1.87)*	1.40 (1.03–1.91)*	1.79 (1.25–2.57)**	1.42 (1.08–1.87)*
Emotional Support	0.87 (0.46–1.66)	0.90 (0.50–1.60)	0.84 (0.45–1.58)	0.79 (0.43–1.44)
Service Attendance	0.66 (0.45–0.98)*	0.67 (0.45–0.99)*	0.67 (0.45–0.99)*	0.66 (0.44–0.99)*
Gender				
Men^a^	--	--	--	--
Women	2.77 (0.78–9.85)	3.05 (0.83–11.17)	2.79 (0.82–9.53)	2.73 (0.78–9.59)
Age	0.97 (0.91–1.02)	0.96 (0.91–1.02)	0.96 (0.91–1.02)	0.96 (0.91–1.02)
Education	1.07 (0.90–1.26)	1.07 (0.90–1.27)	1.06 (0.89–1.26)	1.07 (0.90–1.25)
Family Income	0.64 (0.32–1.30)	0.68 (0.34–1.36)	0.71 (0.33–1.52)	0.64 (0.31–1.31)
Marital Status				
Married/Cohabiting^a^	--	--	--	--
Separated/Divorced/Widowed	0.75 (0.26–2.21)	0.72 (0.24–2.10)	0.75 (0.24–2.31)	0.76 (0.26–2.26)
Never Married	2.76 (0.63–12.08)	2.81 (0.62–12.61)	2.86 (0.69–11.92)	2.70 (0.61–11.88)
Region				
South^a^	--	--	--	--
Northeast	1.76 (0.43–7.25)	1.78 (0.44–7.11)	1.81 (0.47–6.91)	1.77 (0.41–7.53)
North Central	2.59 (1.00–6.71)*	2.69 (1.03–7.03)*	2.54 (0.92–7.03)	2.56 (0.97–6.75)
West	0.39 (0.58–2.68)	0.46 (0.52–3.98)	0.37 (0.04–3.44)	0.37 (0.06–2.42)
Chronic Health Conditions	1.41 (1.22–1.64)***	1.43 (1.21–1.70)***	1.44 (1.22–1.72)***	1.42 (1.22–1.64)***
Racial Discrimination*Subjective Closeness	--	0.96 (0.94–0.99)*	--	--
Racial Discrimination*Frequency of Contact	--	--	0.98 (0.96–0.99)*	--
Racial Discrimination*Emotional Support	--	--	--	1.01 (0.97–1.05)
*N*	634	634	634	634

AOR=adjusted odds ratio; 95% CI=95% confidence interval;

a Reference category.

* *p* < .05;

** *p*< .01;

*** *p* < .001

**Table 3.** Negative binomial regression analysis for number of 12-month DSM-IV disorders among older African Americans

	Model 1	Model 2	Model 3	Model 4
	B (SE)	B (SE)	B (SE)	B (SE)
Racial Discrimination	0.05 (0.01)**	0.21 (0.06)**	0.17 (0.06)**	0.03 (0.06)
Subjective Closeness	−0.34 (0.27)	0.16 (0.35)	−0.36 (0.26)	−0.35 (0.26)
Frequency of Contact	0.22 (0.11)*	0.23 (0.12)	0.52 (0.15)**	0.23 (0.11)*
Emotional Support	−0.03 (0.28)	−0.003 (0.24)	−0.06 (0.27)	−0.10 (0.35)
Service Attendance	−0.38 (0.20)	−0.36 (0.18)	−0.34 (0.18)	−0.38 (0.20)
Gender				
Men^a^	--	--	--	--
Women	1.42 (0.55)*	1.41 (0.57)*	1.41 (0.52)*	1.42 (0.55)*
Age	−0.04 (0.02)*	−0.04 (0.02)*	−0.04 (0.02)*	−0.04 (0.02)*
Education	0.01 (0.06)	0.01 (0.06)	0.004 (0.06)	0.01 (0.06)
Family Income	−0.29 (0.28)	−0.27 (0.24)	−0.20 (0.29)	−0.28 (0.28)
Marital Status				
Married/Cohabiting^a^	--	--	--	--
Separated/Divorced/Widowed	0.06 (0.50)	0.09 (0.47)	0.06 (0.51)	0.06 (0.51)
Never Married	1.12 (0.60)	1.17 (0.61)	1.13 (0.53)*	1.11 (0.58)
Region				
South^a^	--	--	--	--
Northeast	0.44 (0.62)	0.57 (0.59)	0.37 (0.60)	0.43 (0.62)
North Central	0.55 (0.41)	0.57 (0.40)	0.48 (0.44)	0.53 (0.43)
West	−1.56 (0.90)	−1.41 (1.01)	−1.70 (1.14)	−1.61 (0.92)
Chronic Health Conditions	0.42 (0.11)**	0.42 (0.10)***	0.45 (0.12)**	0.42 (0.11)**
Racial Discrimination*Subjective Closeness	--	−0.05 (0.02)*	--	--
Racial Discrimination*Frequency of Contact	--	--	−0.03 (0.01)*	--
Racial Discrimination*Emotional Support	--	--	--	0.01 (0.02)
Intercept	−0.08 (3.17)	−1.81 (3.10)	−2.25 (3.30)	0.11 (2.87)
*F*	13.05***	10.69***	4.79**	12.24***
Complex Design df	33	33	33	33
*N*	634	634	634	634

B=regression coefficient; SE=standard error;

a Reference category.

Note: Significance test of the individual parameter estimates were based on a complex design-corrected *t*-test.

* *p* < .05;

** *p*< .01;

*** *p* < .001
